# Evaluation of zoonotic platyhelminthe infections identified in slaughtered livestock in Iran, 2015–2019

**DOI:** 10.1186/s12917-021-02888-9

**Published:** 2021-05-05

**Authors:** Behzad Kiani, Christine M. Budke, Ebrahim Shams Abadi, Soheil Hashtarkhani, Amene Raouf Rahmati, Mostafa AkbarPour, Mehdi Zarean, Bibi Razieh Hosseini Farash, Fatemeh Kiani, Elham Moghaddas

**Affiliations:** 1 Department of Medical Informatics, School of Medicine, Mashhad University of Medical Sciences, Mashhad, Iran; 2 Department of Veterinary Integrative Biosciences, Texas A&M University, College Station, TX USA; 3 Department of Organic Chemistry, Faculty of Basic Science, Sabzevar Azad University, Sabzevar, Iran; 4 Department of Parasitology and Mycology, School of Medicine, Mashhad University of Medical Sciences, Mashhad, Iran; 5 Department of Biology, Faculty of Basic Sciences, Imam Hossein University, Tehran, Iran

**Keywords:** Echinococcosis, Dicrocoeliasis, Fascioliasis, Livestock, Spatial analysis, Geographical information systems

## Abstract

**Background:**

Platyhelminth infections of livestock can result in considerable economic losses. This study aims to evaluate the spatial frequency of cystic echinococcosis (CE), dicrocoeliasis, and fascioliasis in livestock slaughtered in Iran during the years 2015–2019 and estimate direct costs associated with organ condemnation due to these parasites.

**Methods:**

Abattoir data from 413 abattoirs representing all 31 Iranian provinces were collected from the Iran Veterinary Organization. Infection prevalence was calculated per year at the province level. The Local Moran’s *I* statistic was performed to evaluate spatial autocorrelation of animals positive at slaughter for the years 2015–2019. Direct costs associated with condemned livers were calculated for each parasitic condition, with costs associated with condemned lungs also included for CE.

**Results:**

Overall prevalence values for the study timeframe were as follows: sheep and goat fascioliasis 1.56% (95% CI: 1.56–1.56%), cattle fascioliasis 3.86% (95% CI: 3.85–3.88%), sheep and goat dicrocoeliasis 4.63% (95% CI: 4.62–4.63%), cattle dicrocoeliasis 3.08% (95% CI: 3.07–3.09%), sheep and goat CE 5.32% (95% CI: 5.32–5.33%), and cattle CE 7.26% (95% CI: 7.24–7.28%). Northwest Iran had the highest prevalence of CE and fascioliasis. High infection areas for *Dicrocoelium* spp. included the provinces of Zanjan, Gilan, Qazvin, and Tehran, which are located in northern Iran. Direct economic losses for sheep and goat fascioliasis, dicrocoeliasis, and CE for the study period were US$13,842,759, US$41,771,377, and US$22,801,054, respectively. Direct economic losses for cattle fascioliasis, dicrocoeliasis, and CE for the study period were US$1,989,200, US$1,668,986, and US$2,656,568, respectively.

**Conclusion:**

Our findings provide valuable data for future monitoring of these important parasitic diseases in Iranian livestock. Disease control strategies are required to reduce the economic and public health impact of these platyhelminths.

**Supplementary Information:**

The online version contains supplementary material available at 10.1186/s12917-021-02888-9.

## Background

Livestock products provide an important source of protein for people worldwide [1]. Helminth infections are common in livestock and can result in economic losses to the livestock industry [2, 3]. Parasite infections can lead to decreased growth, weight, and fertility, which can impact the production and/or quality of meat, milk, and hide/wool [4, 5]. For example, financial losses due to fascioliasis in livestock are estimated to total US$ 3 billion per year globally [6].

Fascioliasis and dicrocoeliasis are two of the most common zoonotic helminth diseases of domestic livestock and both are included on the World Health Organization’s (WHO) list of important human foodborne infections [7, 8]. These trematodes are found in the gallbladder and bile ducts of ruminants such as sheep, cattle, and goats [9]. Humans can also become infected via inadvertent ingestion of metacercariae on aquatic plants (*Fasciola* spp.) or in ants (*Dicrocoelium* spp.) [10, 11]. A systematic review estimated a fascioliasis pooled prevalence of 6.2% (95% Confidence Interval (CI): 5.8–6.5%) in Iranian livestock, with prevalence values of 3.1% (95% CI: 2.4–3.7%) in goats, 4.2% (95% CI: 3.8–4.5%) in sheep, and 9.0% (95% CI: 8.0–9.9%) in cattle [12]. In northern Iran, a large outbreak of human fascioliasis occurred in 1989, infecting approximately 10,000 individuals in Gilan Province. In 1999, another outbreak occurred in the same region, infecting approximately 5000 people. Also, in 2000, there were a reported 1306 cases of human fascioliasis in the province of Gilan [13].

Dicrocoeliasis is known to be quite common in Iranian livestock. Between 2015 and 2018, 2.9% of 571,991 slaughtered sheep and goats and 0.8% of 80,001 slaughtered cattle were positive for *Dicrocoelium dendriticum* in Tehran Province [14]. Human dicrocoeliasis occurs sporadically in Iran, with only five cases reported in the literature [15].

Cystic echinococcosis (CE), due to infection with the larval form of *Echinocococcus granulosus* sensu lato, is a globally important disease caused by zoonotic cestodes that cycle between canid definitive hosts and a variety of livestock intermediate hosts. Humans can act as aberrant intermediate hosts through ingestion of parasite eggs found on infected dogs, contaminated vegetables, or in water [16]. Hydatid cysts can be observed in various parts of the body, but are most commonly found in the liver and lungs [17]. CE is considered an endemic disease in Iran, especially in rural communities [18]. Between 1995 and 2014, 8518 cases of human CE were recorded in different provinces of Iran, with the largest number of cases from Razavi Khorasan Province. The average annual number of human cases was 277 for this time period [19]. Between 1990 and 2015, a study found that 5.9% of sheep, 8.8% of cattle, and 6.4% of goats in Iran had CE at the time of slaughter [20]. An additional study conducted in Tehran Province, found that 2.5% of 571,991 slaughtered sheep and 2.2% of 80,001 slaughtered cattle had CE between February 1, 2015, and January 31, 2018 [14]. The objective of the current study was to determine the province-level prevalence, spatial distribution, and direct economic impact of livestock infections with *Fasciola* spp., *Dicrocoelium* spp., and *E. granulosuss* s.l. in Iran for the years 2015–2019.

## Methods

### Data collection and infection prevalence

**Fig. 1:**
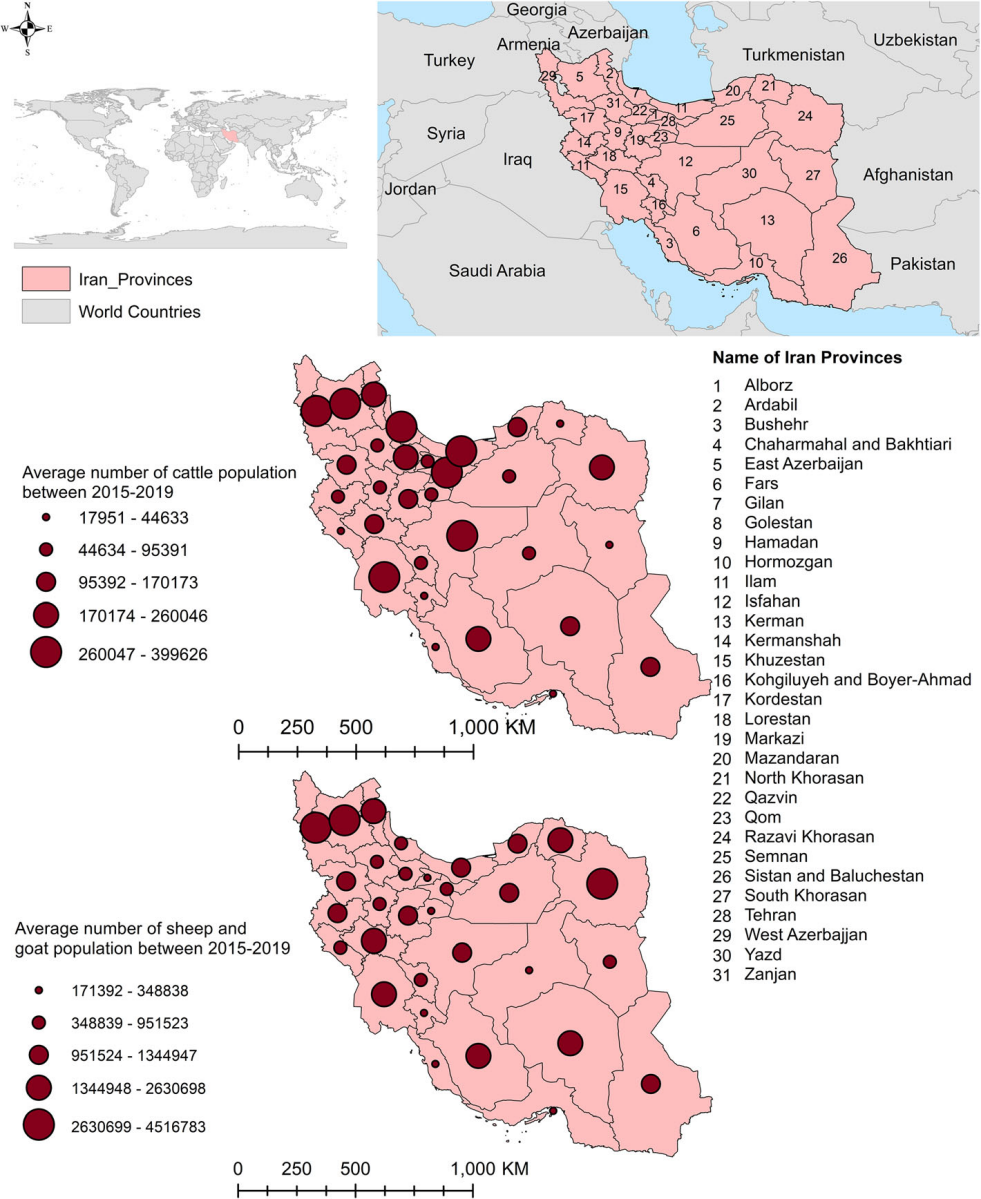
Average number of livestock (cattle, sheep, and goats) per year in 31 Iranian provinces, 2015–2019 (https://www.amar.org.ir)

Sheep are the most common livestock in Iran, followed by goats, and cattle. In April 2020, there were approximately 4,900,000 cattle, 47,300,000 sheep, and 17,000,000 goats in Iran (https://www.amar.org.ir), with the highest livestock populations in the northwestern part of the country (Fig. 1) [21]. This retrospective study was conducted by using data from the Iran Veterinary Organization, which is a central repository for all abattoir-based data. In the current study, data from 2015 to 2019 were assessed from 413 abattoirs representing all 31 Iranian provinces. The data included total number of slaughtered cattle, sheep, and goats by abattoir location as well as fascioliasis, dicrocoeliasis, and CE infection status. Since these three parasites are visible by the naked eye on postmortem inspection, detection was carried out by official veterinary meat inspectors working at the various abattoirs. There have been no studies conducted in Iran to evaluate the sensitivity and specificity of the meat inspection process to detect platyhelminthes. However, a study conducted in Switzerland reported a sensitivity of 63.2% (95% credible interval: 55.6–70.6%) and a specificity nearing 100% to detect liver flukes in cattle using visual meat inspection [22]. A study conducted in Australia found a sensitivity of 24.9% (95% CI: 18.9–32.3%) and a specificity of 98.9% (95% CI: 97.6–99.6%) to detect CE in cattle using standard visual meat inspection practices [23]. Infection prevalence was obtained by evaluating the number of positive animals divided by the number of animals slaughtered per year (Additional File 1). Confidence intervals were calculated by Wilson method.

### Spatial analyses

Descriptive maps of the prevalence of animals infected with *Dicrocoelium spp., Fasciola* spp., and *E. granulosus* s.l. were created for the years 2015–2019 using natural break classification, with five classes. Natural break classification is a data grouping method designed to determine the best arrangement of values into different classes. This is conducted by seeking to minimize each class’s average deviation from the class mean, while maximizing each class’s deviation from the means of the other groups. In other words, the method seeks to reduce the variance within classes and maximize the variance between classes [24].

The Local Moran’s *I* statistic was performed to quantify spatial autocorrelation of prevalence at the provincial level. The number of 499 permutations was used during analysis. This test calculates a z-score and *p*-value to determine whether the apparent similarity (a spatial clustering of either high or low values) or dissimilarity (a spatial outlier) is more pronounced than one would expect in a random distribution. The null hypothesis states that prevalence values are randomly distributed across the study area. High-high and low-low regions indicate that the target area is encompassed by regions with similar prevalence values, while high-low and low-high regions show that the target area is encompassed by regions with dissimilar prevalence values. In other words, the high-high and low-low areas indicate clusters of prevalence at a certain level [25]. ArcGIS 10.5 was used for space-time analyses.

### Economic impact

Direct costs associated with offal condemnation due to fascioliasis (liver), dicrocoeliasis (liver), and CE (liver and/or lungs) were estimated. An animal with CE cysts in both the liver and lungs would contribute both organs to the economic estimate, but would only be considered once for evaluation of CE prevalence. The average price of liver and lungs (cattle liver = US$8.20, sheep/goat liver = US$15.24, cattle lung = US$1.76, and sheep/goat lung = US$0.70) were acquired from the Meisam abattoir in Tehran for the years 2015–2019, with the assumption that there is little variation in price countrywide. The prices of livers and lungs were then multiplied by the number of condemned organs per year (Additional file 2). Costs were converted from the Iranian rial to the US$ for each study year using the free exchange rate (https://en.wikipedia.org/wiki/Iranian_rial).

## Results

### Parasite infection prevalence

**Fig. 2:**
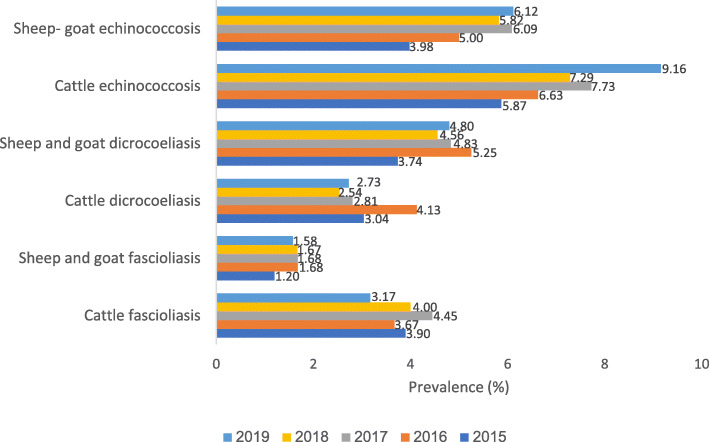
Annual prevalence of fascioliasis, dicrocoeliasis, and CE in livestock slaughtered in 31 Iranian provinces, 2015–2019

In total, 3.86% (95% CI: 3.85–3.88%) of slaughtered cattle and 1.56% (95% CI: 1.56–1.56%) of slaughtered sheep and goats where infected with *Fasciola* spp*.* from 2015 to 2019. Annual prevalence of cattle fascioliasis ranged from 3.2% in 2019 to 3.9% in 2015, while the annual prevalence of sheep and goat fascioliasis ranged from 1.2% in 2015 to 1.6% in 2019 (Fig. 2). The highest number of cases of fascioliasis where in the provinces of Ardabil and East Azerbaijan, which are located in northwestern Iran (Additional File 3).

In total, 3.08% (95% CI: 3.07–3.09%) of slaughtered cattle and 4.63% (95% CI: 4.62–4.63%) of slaughtered sheep and goats where infected with *Dicrocoelium* spp*.* from 2015 to 2019. Annual prevalence of cattle dicrocoeliasis ranged from 2.7% in 2019 to 3.0% in 2015, while the annual prevalence of dicrocoeliasis in sheep and goats ranged from 3.7% in 2015 to 4.8% in 2019 (Fig. 2). The majority of cases of dicrocoeliasis were found in northern Iran (Additional File 4).

In total, 7.26% (95% CI: 7.24–7.28%) of slaughtered cattle and 5.32% (95% CI: 5.32–5.33%) of slaughtered sheep and goats where infected with *E. granulosus* s.l. from 2015 to 2019. Annual prevalence of cattle CE ranged from 5.9% in 2015 to 9.2% in 2019, while the annual prevalence in sheep and goats ranged from 4.0% in 2015 to 6.1% in 2019 (Fig. 2). The majority of CE cases were found in northern Iran (Additional File 5).

### Spatial analysis

**Fig. 3:**
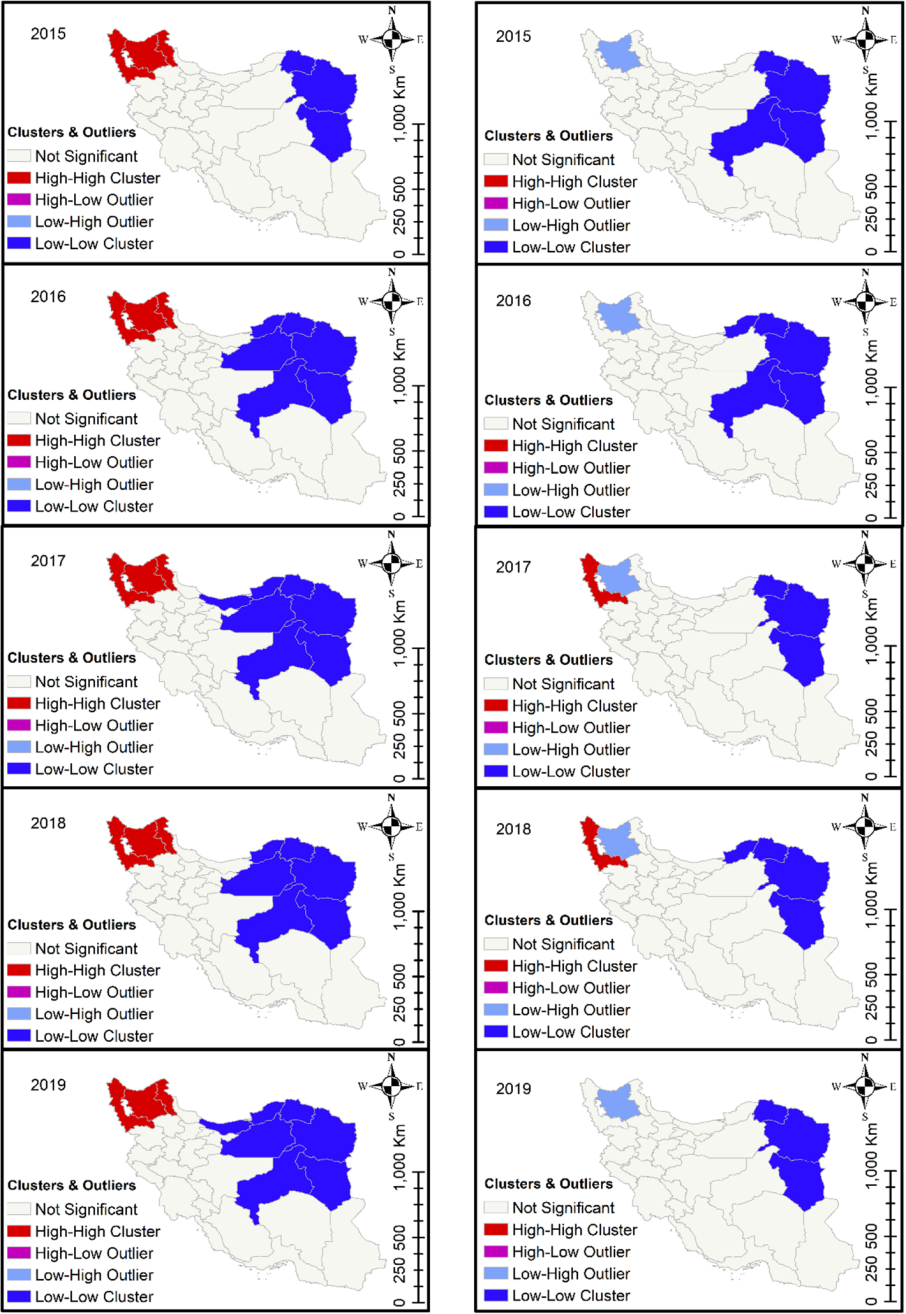
Cluster analysis of fascioliasis in cattle (left panels) and in sheep and goats (right panels) in 31 Iranian provinces, 2015–2019

**Fig. 4:**
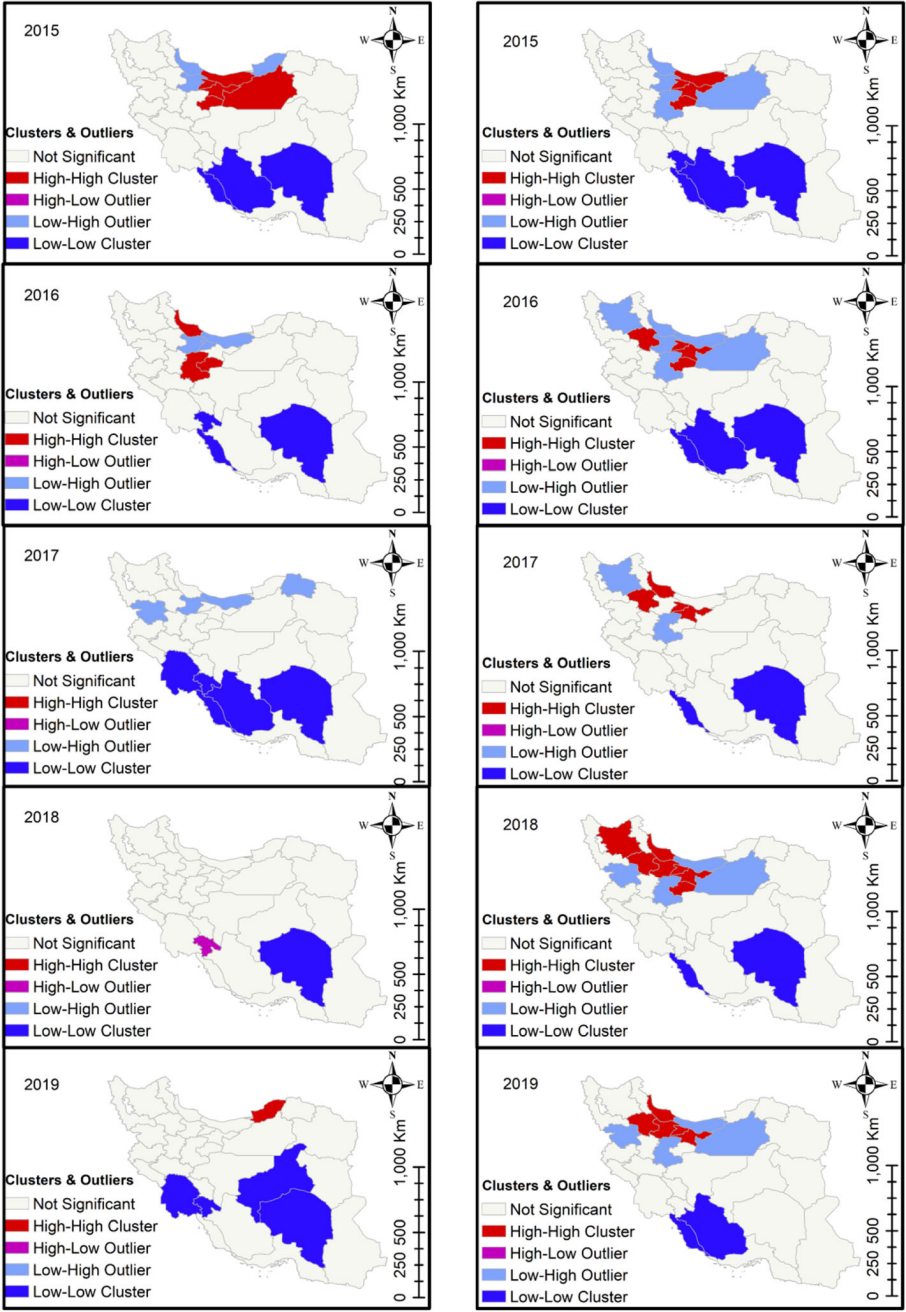
Cluster analysis of dicrocoeliasis in cattle (left panels) and in sheep and goats (right panels) in 31 Iranian provinces, 2015–2019

**Fig. 5:**
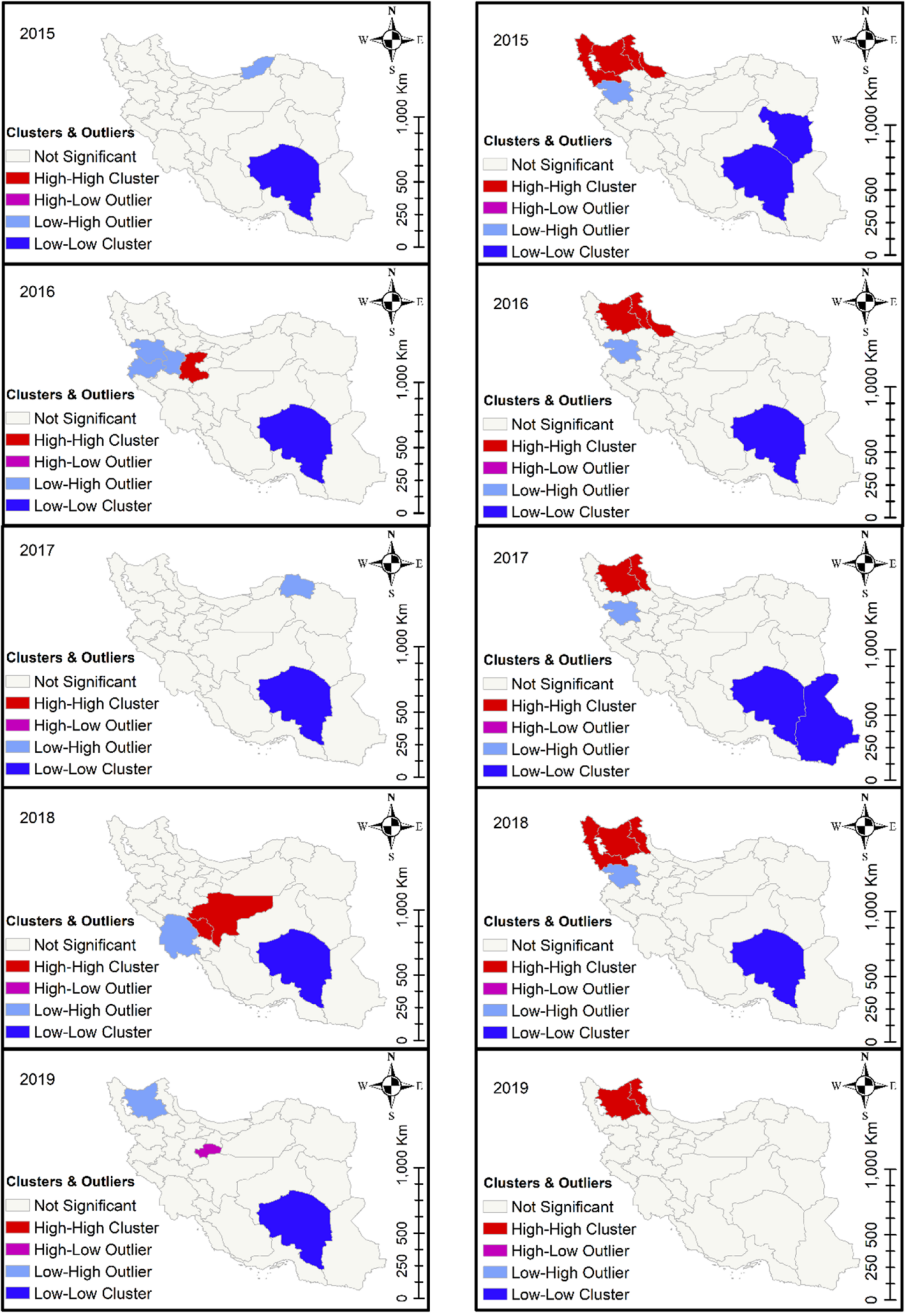
Cluster analysis of CE in cattle (left panels) and in sheep and goats (right panels) in 31 Iranian provinces, 2015–2019

The provinces of East Azerbaijan, West Azerbaijan, and Ardabil, which are all located in northwestern Iran, exhibited high-high cluster patterns for cattle fascioliasis. East Azerbaijan was also identified as a high-risk area for sheep and goat fascioliasis (Fig. 3). Dicrocoeliasis was most common in the northern provinces of Zanjan, Gilan, Qazvin, and Tehran (Fig. 4). The northern provinces of Isfahan, Chaharmahal, and Bakhtiari exhibited high-high cluster patterns for cattle CE, while sheep and goat CE was common in northwestern Iran (Fig. 5). The local Moran’s index values and *P*-values for the maps presented in Figs. 3, 4 and 5 are shown in Additional File 6.

### Economic impact

A total of 233,175 cattle livers and 910,282 sheep and goat livers were discarded due to fascioliasis during 2015–2019. These losses resulted in direct cost estimates of US$ 1,989,200 for cattle and US$ 13,842,759 for sheep and goats. During this same time period, 186,009 cattle livers and 2,701,274 sheep and goat livers where condemned due to dicrocoeliasis, resulting in losses of US$ 1,668,986 and US$ 41,771,377, respectively. In addition, 311,982 cattle livers and 1,500,385 sheep and goat livers were discarded due to CE, resulting in losses of US$2,422,035 and US$21,639,272**,** respectively. Furthermore, 126,602 cattle lungs and 1,608,382 sheep and goat lungs were discarded due to CE, resulting in losses of US$234,532 and US$1,161,781, respectively. These condemnations resulted in an estimated direct loss of US$84,729,943 due to the three parasitic infections over the five-year period (Additional File 7).

## Discussion

This is the first spatial analysis of fascioliasis, dicrocoeliasis, and CE prevalence in slaughtered livestock for the entire country of Iran. Slaughtered cattle and small ruminants were more commonly infected with CE compared to the other evaluated parasites during the study timeframe. CE is endemic in the Middle East, with a recent systematic review calculating a weighted prevalence of 15.6% (95% CI = 14.2–17.1%) in livestock (cattle, sheep, goats, buffalos, camels, donkeys, and boars) and 4.2% (95% CI = 3.0–5.5%) in humans from Iran [20]. The mean annual prevalence values for CE in cattle (5.3%) and sheep and goats (7.3%) reported in the current study align with findings from Khalkhali et al. (2018) who reported a prevalence of 8.8% in cattle, 5.9% in sheep, and 6.4% in goats in Iran [20]. We identified high CE prevalence values in the northwestern region of the country, with much lower values in southern Iran. These distributions align with reported high prevalence values for human CE in northern (9, 95% CI: 4–18%) and western (6, 95% CI: 3–11%) Iran [26].

Porous international borders make it difficult to control illegal importation of livestock infected with CE, especially when neighboring countries continue to have problems with the parasite. CE prevalence in cattle and small ruminants was found to be 8.0 and 8.9%, respectively, in Pakistan [27]. In Iraq, 30% of cattle and 32% of small ruminants were found to be infected with CE [28]. Very limited information is currently available from Afghanistan and Turkmenistan. However, they are both also believed to be endemic for *E. granulosus* s.l. [29].

Dicrocoeliasis was the second most prevalent parasite found in this study, which aligns with other abattoir-based surveys conducted in Iran [30, 31]. It is believed that *Dicrocoelium* spp. eggs can survive for a longer time on pastures compared to the eggs of *Fasciola* spp. Based on the data presented here, high infection areas for *Dicrocoelium* spp. were identified in northern Iran. A study focused on predicting the spatial distribution and environmental suitability for *Dicrocoelium* spp. identified the littoral of the Caspian Sea as a high-risk area, with regional prevalence values of 36.7 and 6.1% for sheep and cattle, respectively [32]. A temperature range of 10–25 °C and high levels of moisture are needed for the development of both *Fasciola* larvae and its intermediate snail host (*Lymnaea truncatula*) [33]. Over the past decade, Iran has experienced a prolonged and severe drought [34]. At present, the northern, northwestern, and western regions of Iran appear to be most suitable for completion of the *Fasciola* spp. life cycle [12, 35, 36]. Spatial analysis confirmed that northwest Iran was a high prevalence region for fascioliasis. Use of spatial analysis is helpful to highlight regions of concern and to help plan control initiatives.

CE (liver and/or lung infection) was the most prevalent evaluated parasitic infection in slaughtered Iranian livestock, but economic losses were greatest for dicrocoeliasis since the price of liver is approximately 12 times the price of lung. Although few economic assessments have been performed to evaluate costs due to dicrocoeliasis, *D. dendriticum* was responsible for 26% of marketable organ condemnation due to parasite infection in the region of Trikala, Greece. However, since only a small percentage (0.26%) of organs were condemned due to parasitic infections in this region of Greece, the economic impact was negligible [37]. Annual economic losses due to cattle fascioliasis in Iran were estimated to be 28.9 and 9.2 times the losses recorded in Nigeria (US$13,773) and Ethiopia (US$43,024), respectively [38, 39]. Annual economic losses due to cattle CE were estimated to be 9.1 times the losses in Ethiopia (US$ 58,114) and 7.3 times the losses in Australia (US$ 66,893) [40, 41].

The present study did have a number of limitations. First, we were unable to account for mixed infections with more than one parasite. This is because aggregated data received from the Iran Veterinary Organization did not include the number of animals with more than one parasite in a specific data field. Instead, if an animal carried more than one parasite, it was counted in all related data fields (Additional File 1). In other words, this limitation did not affect our maps but it did not allow us to create a hybrid map (e.g. a map containing both *Dicrocoelium spp.* and *Fasciola spp.*).

Second, we were not able to analyze sheep and goat data separately since sheep and goat slaughter lines are not differentiated in Iran. In addition, CE cysts located in organs other than the liver and lungs were likely missed. Therefore, prevalence values may be underestimated. An updated reporting structure is needed to be able to better assess parasite frequency and impacted livestock species. Also, only direct costs due to organ condemnation were evaluated. These parasitic conditions likely have additional impacts on animal growth and other production measures, which were not assessed in this study. Overall, the prevalence of these three parasites appears to have either remained steady or increased (e.g., cattle CE) between 2015 and 2019. Since these parasites are potential human health hazards, special attention should be given to their control [42].

## Conclusion

This study has shown that platyhelminth infections continue to be prevalent in Iranian livestock and result in substantial economic losses. These values reinforce the necessity of developing effective control programs based on regional needs.

## Supplementary Information

**Additional file 1.**

**Additional file 2.**

**Additional file 3.**

**Additional file 4.**

**Additional file 5.**

**Additional file 6.** (XLS 148 kb)

**Additional file 7.**

## Data Availability

The datasets used and analysed during the current study are available to the public from the Additional File 1.

## Abbreviations

CE: Cystic echinococcosis
CI: Confidence interval

## References

[1] Grigg D (1995). The pattern of world protein consumption. Geoforum.

[2] Arbabi M, Nezami E, Hooshyar H, Delavari M (2018). Epidemiology and economic loss of fasciolosis and dicrocoeliosis in Arak, Iran. Veterinary world.

[3] Charlier J, Rinaldi L, Musella V, Ploeger HW, Chartier C, Vineer HR, Hinney B, von Samson-Himmelstjerna G, Băcescu B, Mickiewicz M (2020). Initial assessment of the economic burden of major parasitic helminth infections to the ruminant livestock industry in Europe. Prev Vet Med.

[4] Umur S, Kaaden OR (2003). Prevalence and economic importance of cystic Echinococcosis in slaughtered ruminants in Burdur, Turkey 1. J Veterinary Med Ser B.

[5] Charlier J, van der Voort M, Kenyon F, Skuce P, Vercruysse J (2014). Chasing helminths and their economic impact on farmed ruminants. Trends Parasitol.

[6] Elliott T, Kelley J, Rawlin G, Spithill T (2015). High prevalence of fasciolosis and evaluation of drug efficacy against Fasciola hepatica in dairy cattle in the Maffra and Bairnsdale districts of Gippsland, Victoria, Australia. Vet Parasitol.

[7] Organization WH (2011). Report of the WHO expert consultation on foodborne trematode infections and taeniasis. World Health Organization.

[8] Organization WH (2007). Initiative to estimate the global burden of foodborne diseases. First formal meeting of the Foodborne Disease Burden Epidemiology Reference Group. Geneva.

[9] Youn H (2009). Review of zoonotic parasites in medical and veterinary fields in the Republic of Korea. Korean J Parasitol.

[10] SABZEVARINEZHAD G (2004). Flukes Liver Epidemic Common Between Human and Livestock in Slaughtered and Their Staining.

[11] Otranto D, Traversa D (2002). A review of dicrocoeliosis of ruminants including recent advances in the diagnosis and treatment. Vet Parasitol.

[12] Khademvatan S, Majidiani H, Khalkhali H, Taghipour A, Asadi N, Yousefi E (2019). Prevalence of fasciolosis in livestock and humans: a systematic review and meta-analysis in Iran. Comp Immunol Microbiol Infect Dis.

[13] Ashrafi K (2015). The status of human and animal fascioliasis in Iran: a narrative review article. Iran J Parasitol.

[14] Pezeshki A, Aminfar H, Aminzare M (2018). An analysis of common foodborne parasitic zoonoses in slaughtered sheep and cattle in Tehran, Iran, during 2015-2018. Veterinary World.

[15] Ashrafi K (2010). Human dicrocoeliasis in northern Iran: two case reports from Gilan province. Ann Trop Med Parasitol.

[16] Rokni M. Echinococcosis/hydatidosis in Iran. Iran J Parasitol. 2009;4(2):1–16.

[17] Jenkins D, Romig T, Thompson R (2005). Emergence/re-emergence of Echinococcus spp.—a global update. Int J Parasitol.

[18] Hashemnia M, Safavi EAA (2016). A retrospective survey of hydatidosis based on abattoir data in Kermanshah, Iran from 2008 to 2013. J Parasit Dis.

[19] Zeinali M, Mohebali M, Shirzadi MR, ESBOEI BR, Erfani H, Pourmozafari J, Ghanbari M (2017). Human cystic Echinococcosis in different geographical zones of Iran: an observational study during 1995–2014. Iran J Public Health.

[20] Khalkhali H, Foroutan M, Khademvatan S, Majidiani H, Aryamand S, Khezri P, Aminpour A (2018). Prevalence of cystic echinococcosis in Iran: a systematic review and meta-analysis. J Helminthol.

[21] Amiraslani F, Dragovich D (2011). Combating desertification in Iran over the last 50 years: an overview of changing approaches. J Environ Manag.

[22] Rapsch C, Schweizer G, Grimm F, Kohler L, Bauer C, Deplazes P, Braun U, Torgerson PR (2006). Estimating the true prevalence of Fasciola hepatica in cattle slaughtered in Switzerland in the absence of an absolute diagnostic test. Int J Parasitol.

[23] Wilson CS, Jenkins DJ, Barnes TS, Brookes VJ (2019). Evaluation of the diagnostic sensitivity and specificity of meat inspection for hepatic hydatid disease in beef cattle in an Australian abattoir. Prev Vet Med.

[24] ESRI Data classification methods. https://pro.arcgis.com/en/pro-app/help/mapping/layer-properties/data-classification-methods.htm. Accessed 28 November 2020.

[25] Moran P (1950). Notes on continuous stochastic phenomena. Biometrika.

[26] Khademvatan S, Majidiani H, Foroutan M, Tappeh KH, Aryamand S, Khalkhali H (2019). Echinococcus granulosus genotypes in Iran: a systematic review. J Helminthol.

[27] Mehmood N, Arshad M, Ahmed H, Simsek S, Muqaddas H (2020). Comprehensive account on prevalence and characteristics of Hydatid cysts in livestock from Pakistan. Korean J Parasitol.

[28] Alsaady HAM, Al-Quzweeni HAN (2019). Molecular study of Echinococcus Granulosus in Misan Province, south of Iraq. Indian J Public Health Res Dev.

[29] Zhang W, Zhang Z, Wu W, Shi B, Li J, Zhou X, Wen H, McManus DP (2015). Epidemiology and control of echinococcosis in Central Asia, with particular reference to the People's Republic of China. Acta Trop.

[30] Najjari M, Karimazar M, Rezaeian S, Ebrahimipour M, Faridi A (2020). Prevalence and economic impact of cystic echinococcosis and liver fluke infections in slaughtered sheep and goat in north-Central Iran, 2008–2018. J Parasit Dis.

[31] Motazedian M, Najjari M, Zarean M, Karimi G, Karimazar M, Ebrahimipour M (2019). An abattoir survey of hydatid and liver fluke disease in slaughtered cattle in Alborz Province, Iran. Comp Clin Pathol.

[32] Meshgi B, Majidi-Rad M, Hanafi-Bojd AA, Kazemzadeh A (2019). Predicting environmental suitability and geographical distribution of Dicrocoelium dendriticum at littoral of Caspian Sea: an ecological niche-based modeling. Prev Vet Med.

[33] Fox NJ, White PC, McClean CJ, Marion G, Evans A, Hutchings MR (2011). Predicting impacts of climate change on Fasciola hepatica risk. PLoS One.

[34] Emadodin I, Reinsch T, Taube F (2019). Drought and desertification in Iran. Hydrology.

[35] Khanjari A, Bahonar A, Fallah S, Bagheri M, Alizadeh A, Fallah M, Khanjari Z (2014). Prevalence of fasciolosis and dicrocoeliosis in slaughtered sheep and goats in Amol abattoir, Mazandaran, northern Iran. Asian Pac J Trop Dis.

[36] Ezatpour B, Hasanvand A, Azami M, Mahmoudvand H, Anbari K (2014). A slaughterhouse study on prevalence of some helminths of cattle in Lorestan provience, West Iran. Asian Pac J Trop Dis.

[37] Theodoropoulos G, Theodoropoulou E, Petrakos G, Kantzoura V, Kostopoulos J (2002). Abattoir condemnation due to parasitic infections and its economic implications in the region of Trikala, Greece. J Veterinary Med Ser B.

[38] Ola-Fadunsin SD, Uwabujo PI, Halleed IN, Richards B (2020). Prevalence and financial loss estimation of parasitic diseases detected in slaughtered cattle in Kwara state, north-Central Nigeria. J Parasit Dis.

[39] Zewde A, Bayu Y, Wondimu A (2019). Prevalence of bovine Fasciolosis and its economic loss due to liver condemnation at Wolaita Sodo municipal Abattair, Ethiopia. Veterinary Medicine International 2019.

[40] Guduro GG, Desta AH (2019). Cyst viability and economic significance of Hydatidosis in southern Ethiopia. J Parasitol Res.

[41] Wilson CS, Jenkins DJ, Brookes VJ, Barnes TS, Budke CM (2020). Assessment of the direct economic losses associated with hydatid disease (Echinococcus granulosus sensu stricto) in beef cattle slaughtered at an Australian abattoir. Prev Vet Med.

[42] Ekong PS, Juryit R, Dika NM, Nguku P, Musenero M. Prevalence and risk factors for zoonotic helminth infection among humans and animals-Jos, Nigeria, 2005-2009. Pan Afr Med J. 2012;12(1). 10.11604/pamj.2012.12.6.1110. PMC3396872 22826731

