# Supplementary material for: Evaluation of zoonotic platyhelminthe infections identified in slaughtered livestock in Iran, 2015–2019
Source: BMC Vet Res. 2021 May 5;17:185. doi: 10.1186/s12917-021-02888-9 (PMC8097913; doi:10.1186/s12917-021-02888-9)
Supplement: Supplementary file 2 — Additional file 2. [file 12917_2021_2888_MOESM2_ESM.docx]

The prices of livers and lungs from 2015-2019

| Year | Cattle Liver | | Sheep/Goat Liver | | Cattle Lung | | Sheep/Goat Lung | |
| --- | --- | --- | --- | --- | --- | --- | --- | --- |
|  | Iranian  Rial | US  Dollar | Iranian  Rial | US  Dollar | Iranian  Rial | US  Dollar | Iranian  Rial | US  Dollar |
| 2015 | 409,860 | 11.88 | 740,025 | 21.45 | 88,665 | 2.57 | 35,190 | 1.02 |
| 2016 | 459,900 | 12.60 | 820,155 | 22.47 | 98,550 | 2.70 | 39,055 | 1.07 |
| 2017 | 509,920 | 8.57 | 909,755 | 15.29 | 110,075 | 1.85 | 44,030 | 0.74 |
| 2018 | 569,700 | 4.22 | 1,000,350 | 7.41 | 122,850 | 0.91 | 48,600 | 0.36 |
| 2019 | 630,700 | 3.71 | 1,153,250 | 6.59 | 132,600 | 0.78 | 54,400 | 0.32 |
| Average | 516,016 | 8.196 | 924,707 | 15.242 | 110,548 | 1.762 | 44,255 | 0.702 |

Free exchange rates (Iranian rials to US$): 2015- 34,500 rials to 1 US$, 2016- 36,500 rials to 1 US$, 2017- 59,500 rials to 1 US$, 2018- 135,000 rials to 1 US$, and 2019- 170,000 rials to 1 US$ (https://en.wikipedia.org/wiki/Iranian_rial).
