# Supplementary material for: Evaluation of zoonotic platyhelminthe infections identified in slaughtered livestock in Iran, 2015–2019
Source: BMC Vet Res. 2021 May 5;17:185. doi: 10.1186/s12917-021-02888-9 (PMC8097913; doi:10.1186/s12917-021-02888-9)
Supplement: Supplementary file 5 — Additional file 5. [file 12917_2021_2888_MOESM5_ESM.docx]

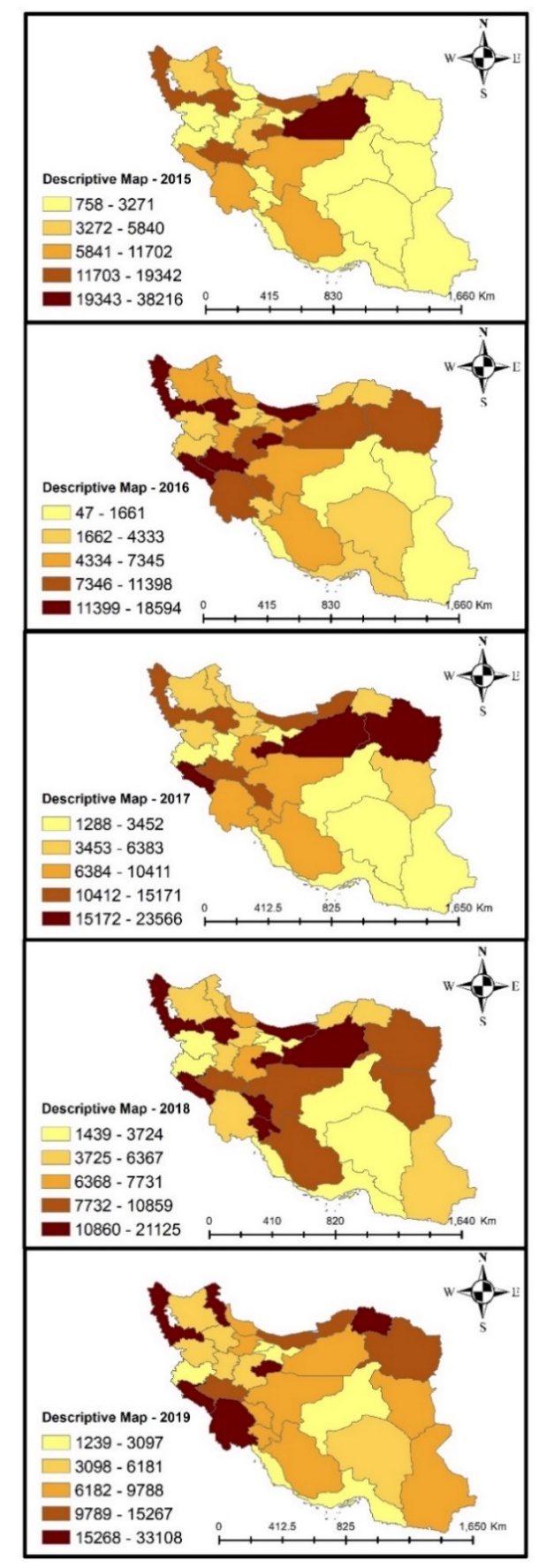

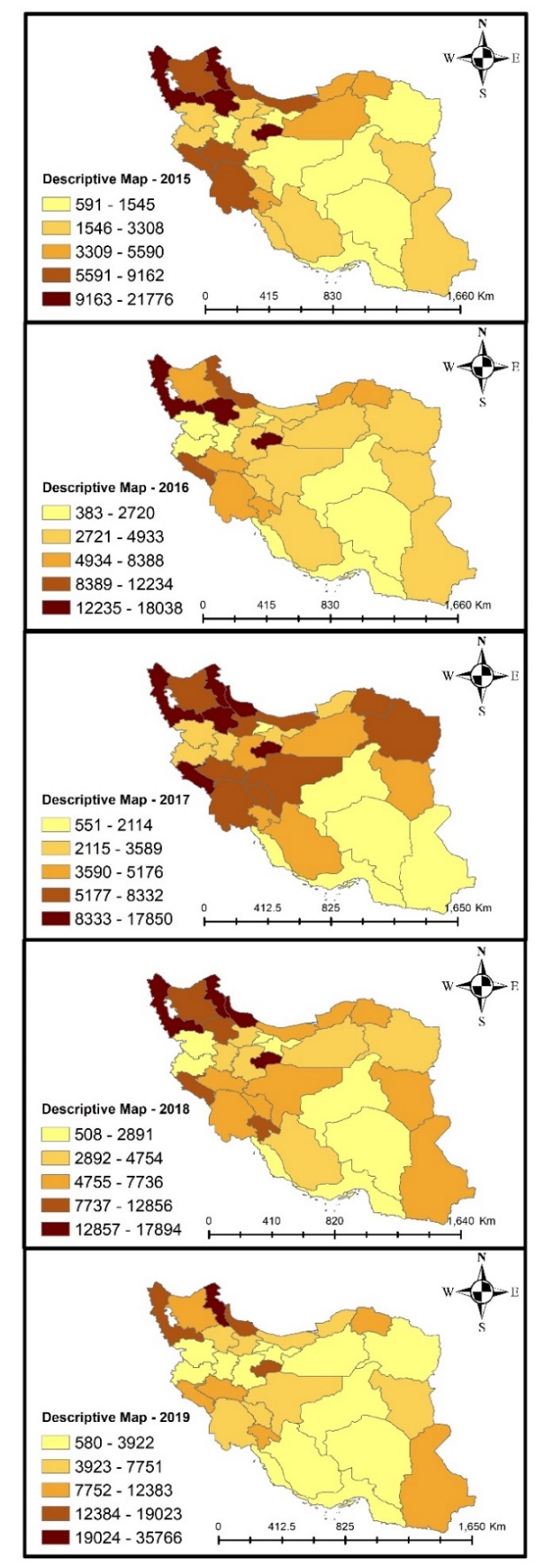


CE infection per 100,000 slaughtered cattle (left panels) and sheep and goats (right panels) in 31 Iranian provinces, 2015-2019.
