# Supplementary material for: Evaluation of zoonotic platyhelminthe infections identified in slaughtered livestock in Iran, 2015–2019
Source: BMC Vet Res. 2021 May 5;17:185. doi: 10.1186/s12917-021-02888-9 (PMC8097913; doi:10.1186/s12917-021-02888-9)
Supplement: Supplementary file 7 — Additional file 7. [file 12917_2021_2888_MOESM7_ESM.docx]

Direct costs (in US$) associated with livestock infection with fascioliasis, dicrocoeliasis, and CE in 31 Iranian provinces, 2015-2019

| Year | | Cattle fascioliasis | Sheep and goat fascioliasis | Cattle dicrocoeliasis | Sheep and goat dicrocoeliasis | Cattle CE | Sheep and goat CE | Total infections |
| --- | --- | --- | --- | --- | --- | --- | --- | --- |
| 2015 | Number of condemned livers | 50,367 | 158,928 | 39,258 | 495,729 | 47,358 | 233,556 | 1,025,196 |
|  | Number of condemned lungs |  |  |  |  | 28,416 | 293,555 | 321,971 |
|  | Cost (US$) | 598,359 | 3,409,005 | 466,385 | 10,633,387 | 635,642 | 5,309,202 | 21,051,982 |
| 2016 | Number of condemned livers | 47,479 | 222,568 | 53,337 | 695,224 | 47,591 | 290,481 | 1,356,680 |
|  | Number of condemned lungs |  |  |  |  | 38,073 | 372,481 | 410,554 |
|  | Cost (US$) | 598,235 | 5,001,102 | 672,046 | 15,621,683 | 702,444 | 6,925,663 | 29,521,174 |
| 2017 | Number of condemned livers | 54,782 | 206,655 | 34,674 | 592,604 | 86,517 | 393,890 | 1,369,122 |
|  | Number of condemned lungs |  |  |  |  | 8,652 | 353,890 | 362,542 |
|  | Cost (US$) | 469,482 | 3,159,755 | 297,156 | 9,060,915 | 757,457 | 6,284,457 | 20,029,222 |
| 2018 | Number of condemned livers | 47,634 | 182,990 | 30,339 | 497,118 | 66,883 | 294,405 | 1,119,369 |
|  | Number of condemned lungs |  |  |  |  | 19,999 | 340,404 | 360,453 |
|  | Cost (US$) | 201,015 | 1,355,956 | 128,031 | 3,683,644 | 300,445 | 2,304,086 | 7,973,178 |
| 2019 | Number of condemned livers | 32,913 | 139,141 | 28,401 | 420,599 | 63,633 | 288,053 | 972,740 |
|  | Number of condemned lungs |  |  |  |  | 31,412 | 248,052 | 279,464 |
|  | Cost (US$) | 122,107 | 916,939 | 105,368 | 2,771,747 | 260,580 | 1,977,646 | 6,154,387 |
| Total | Number of condemned livers | 233,175 | 910,282 | 186,009 | 2,701,274 | 311,982 | 1,500,385 | 5,843,107 |
|  | Number of condemned lungs |  |  |  |  | 126,552 | 1,608,382 | 1,734,934 |
|  | Cost (US$) | 1,989,200 | 13,842,759 | 1,668,986 | 41,771,377 | 2,656,568 | 22,801,054 | 84,729,943 |

Free exchange rates (Iranian rials to US$): 2015- 34,500 rials to 1 US$, 2016- 36,500 rials to 1 US$, 2017- 59,500 rials to 1 US$, 2018- 135,000 rials to 1 US$, and 2019- 170,000 rials to 1 US$ (https://en.wikipedia.org/wiki/Iranian_rial).
